# A Fiber Optic Sensor for Monitoring the Spectral Alterations and Depth in Ex Vivo and In Vivo Cryosurgery

**DOI:** 10.3390/s23052690

**Published:** 2023-03-01

**Authors:** Aris Ikiades, Ioannis D. Bassukas, Nikolaos Kourkoumelis

**Affiliations:** 1 Department of Physics, University of Ioannina, 45110 Ioannina, Greece; 2 Department of Skin & Venereal Diseases, School of Health Sciences, University of Ioannina, 45110 Ioannina, Greece; 3 Department of Medical Physics, School of Health Sciences, University of Ioannina, 45110 Ioannina, Greece

**Keywords:** optical scattering, optical diffusion, skin, cryosurgery, optical fiber sensors

## Abstract

This article discusses how to monitor the freezing depth during cryotherapy using a fiber optic array sensor. The sensor was used to measure the backscattered and transmitted light from frozen and unfrozen ex vivo porcine tissue and in vivo human skin tissue (finger). The technique exploited the variations in optical diffusion properties of the frozen and unfrozen tissues to determine the extent of freezing. Ex vivo and in vivo measurements yielded comparable results, despite spectral variations attributable to the hemoglobin absorption peak in the human frozen and unfrozen tissues. However, because the spectral fingerprints of the freeze-thaw process in the ex vivo and in vivo experiments were similar, we could extrapolate the maximum depth of freezing. Therefore, this sensor has the potential to be utilized for monitoring cryosurgery in real time.

## 1. Introduction

Cryosurgery is a medical procedure that involves the use of extreme cold to destroy abnormal or diseased tissue. It is also known as cryotherapy. The procedure is typically performed using liquid nitrogen or argon gas to freeze and destroy the targeted tissue. Cryosurgery is used to treat a variety of conditions, including skin lesions, prostate cancer, and cervical dysplasia. It is a minimally invasive procedure and recovery time is generally quick [1].

The physiological mechanisms of cryosurgery involve the freezing and thawing of cells, the constriction of blood vessels, and the oxygen and nutrient deprivation of cells, all of which lead to the destruction of the targeted tissue and the stimulation of the healing process. The destruction of the tissue is a gradual process, as the freezing and thawing cycles cause ice crystals to form and grow within the cells, rupturing the cell membrane [2]. Additionally, the cells in the treated area become oxygen and nutrient deprived, with further damage to the tissue promoting cell death [3]. The freezing process also causes an inflammatory response, which contributes to cell death and the healing process [4].

The crucial parameter, freezing depth—and, therefore, the amount of tissue affected by the cryotherapy—is determined by the duration of freezing and the size of the probe used, without having an objective estimation of the expansion and depth of the “ice ball”. Determining the depth of freezing is necessary to effectively treat the condition without causing excessive damage to surrounding healthy tissue. The depth of freezing can be influenced by a number of factors, including the size and location of the lesion, the type of tissue being treated, and the type of equipment being used. If the freezing is not deep enough, the abnormal cells may not be fully destroyed, and the condition may not be effectively treated. If the freezing is too deep, it can cause significant damage to healthy tissue and potentially result in complications, such as nerve damage, tissue necrosis, or scarring. The depth of freezing is typically monitored by imaging techniques such as ultrasound and MRI [5,6,7]. Other methods include direct temperature measurements utilizing infrared thermography [8]. Finally, imaging techniques such as optical coherent tomography (OCT) [9], vibro-acoustography [10], and electrical impedance tomography [11] have also been reported.

In this study, we demonstrate a technique based on fiber optic array geometry that exploits the differential optical diffusion and spectral properties of frozen/unfrozen ex vivo porcine tissues, in order to monitor the depth of the frozen region in real time. The fiber optic array sensor was subsequently used for in vivo spectra measurements during the thawing process.

## 2. Principle of the Measurement Technique

When water-rich living tissues are frozen quickly with liquid nitrogen, their color changes. They turn pale white for a short time after the cryogen is no longer being used. The production of ice crystals and microbubbles contributes to this phenomenon, along with a temporary constriction of arteries and the subsequent lowering of local blood content. Mie or Rayleigh scattering changes the optical characteristics of the frozen tissues, which are dependent on the size of these microstructures [12]. The differences in the optical properties of frozen and unfrozen tissues can be used to determine the depth of freezing. This is achieved by monitoring the change in intensity of the backscattered light, which is associated with an increase in the number of scatterers in the frozen tissue.

In a previous study [13], we demonstrated a method that exploits variations in the backscattering monochromatic intensity of frozen and unfrozen ex vivo tissue to obtain information on the depth of freezing during cryotherapy using a fiber optic sensor. This method involves measuring both the transmitted and backscattered light from frozen and unfrozen tissue simultaneously, in order to determine the maximum transmission depth and calibrate it with the backscattered light from the two tissue states. We measured and calibrated the transmitted and backscattered intensities of frozen and unfrozen tissues at different skin depths. We used an independent method involving ultrasound to validate the depth of freezing measured from the backscattered intensity detected by the fiber array during thawing.

In the experiments reported here, we used a similar experimental arrangement to the one presented in our previous work. This comprised a modified multiple fiber array sensor that illuminated and detected the backscattered light from frozen and unfrozen ex vivo porcine tissue, as well as in vivo human skin. We used a similar calibration procedure, involving a monochromatic laser light to illuminate the ex vivo tissue at different depths, in order to calibrate the backscattered and transmitted light during thawing. Following calibration, we used the fiber array sensor to measure the spectra of both the ex vivo and in vivo tissues during thawing. This is described in more detail in the following section.

## 3. Calibration Depth in Porcine Ex Vivo Tissue

### 3.1. Optical Transmission and Backscattering Setup and Procedure

When living tissue is rapidly frozen by applying liquid nitrogen, changes to the optical characteristics of in vivo or ex vivo tissue can be observed. These changes are due to the formation of microcrystals and microbubbles, which are formed from cellular fluids and dissolved gases, respectively. Additionally, some temporary blisters and inflammation occur, which persist for some time after treatment. However, the “whitening” observed in ex vivo porcine as well as in living tissue after freezing returns to its normal color with no obvious structural damage. This change in tissue optical properties is used to determine the depth of freezing for in vivo and ex vivo tissues using intensity and spectral fluctuations in backscattered light.

The experimental setup used to determine the depth of freezing in ex vivo porcine tissue was similar to the one presented in our previous work [13]. Specifically, square porcine samples with dimensions of approximately 5 × 5 cm and approximately 3 mm in thickness were positioned vertically on a frame so that they could be accessed from both sides. Backscattering and transmission measurements were taken using a fiber array sensor consisting of six detection fibers arranged on either side of an illumination fiber, which detects the backscattered light from the tissue volume. The central illumination fiber was embedded in the tissue with a syringe and could be positioned at different depths with a micrometer. The transmitted light was detected by another fiber located on the opposite side of the tissue and aligned with the illumination fiber. Liquid nitrogen (LN) was applied to the tissue as a cold jet with a special dispenser used in dermatology. For these experiments, the energy extracted from the tissue was measured by the amount of LN used during freezing, which was approximately 10–15 g, corresponding to approximately 2–3 kilojoules (kJ) of energy extracted from the tissue. The application of LN resulted in freezing a circular section of approximately 20–25 mm in diameter, where the tissue became white and extended inside the tissue as an “ice ball”. The depth of freezing could be determined experimentally by measuring the transmitted intensity at different depths of the illumination fiber and correlating it with the backscattered light reaching the fiber optic sensor array.

The backscatter and transmission light characteristics of frozen/unfrozen tissues change due to the variations in scatterers. In the frozen tissue, backscattered intensity increases and transmitted intensity decreases, gradually returning to their original values during thawing. These optical changes can be used to determine the maximum depth at which backscattered light can be detected from frozen tissue by correlating the two intensities. To measure these changes, a 650 nm laser diode was used to illuminate the porcine tissue through the central illumination fiber of a bespoke multi-channel fiber array sensor. Backscattered and transmitted signals were detected by the individual fibers and recorded by a Labview Data Acquisition System. The fibers used in the sensor were multimode with a glass core and polymer cladding, with a diameter of 250 μm, a numerical aperture (NA) of 0.35, and an inter-fiber separation of 1 mm. During thawing, the transmitted intensity was measured using the fiber located on the opposite side of the tissue and aligned with the illumination fiber. The depth of freezing was determined by measuring the transmitted intensity and backscattered signals at different depths of the illumination fiber inside the tissue and correlating the results. These measurements were verified using an ultrasound imaging system typically used in dermatology [13]. However, it is important to note that obtaining transmission measurements for in vivo experiments is not simple. Consequently, initial results from the ex vivo experiments led to the construction of a fiber array sensor in order to determine the depth of freezing, as discussed below.

### 3.2. Measurement of Frozen Skin Thickness with the Fiber Array Sensor

**Figure 1:**
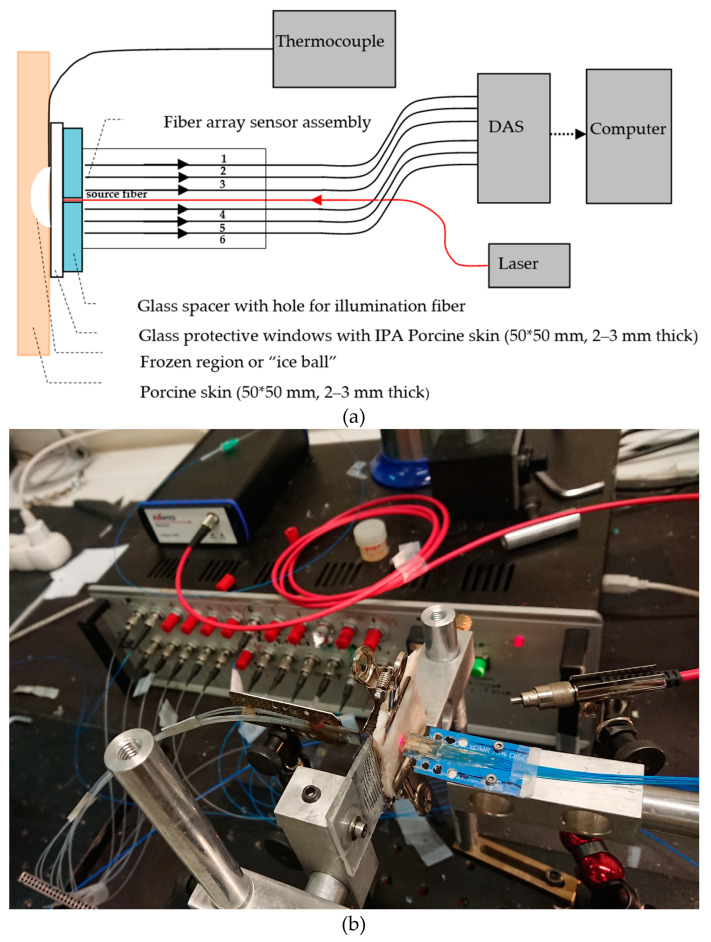
(**a**) Schematic diagram of top view of the fiber array sensor and experimental setup with thermocouple, laser source, and data acquisition system (DAS). (**b**) The fiber array sensor.

The fiber array sensor had the configuration shown in Figure 1, consisting of seven fibers arranged symmetrically on either side of the illumination fiber, which was positioned approximately 2 mm in front of the detection fibers. Several sensor geometries were used in order to measure the optical diffusion and to avoid diffuse reflections from the surface of the frozen and unfrozen tissue. Based on the work in [13], it was decided to switch to MM fibers for both the illumination and detection fibers, with an NA of approximately 0.35. The inter-fiber separation was between 1 mm and 2 mm, and we opted for the latter measurement in this experiment, in order to improve the measurement of optical diffusion, especially in unfrozen cases which extend further in the tissue. This arrangement used a 2 mm glass spacer with a hole, through which the illumination fiber protruded. All the fibers of the array were protected by a second thin glass window, and the assembly was held together with index-matching UV-curing glue, which also provided optical coupling. This optical arrangement was chosen to prevent unwanted diffuse and Fresnel reflections from the tissue surface and from the glass window, respectively, reaching the detection fibers. The sensor was withdrawn momentarily to apply liquid nitrogen to the tissue and then repositioned on the frozen skin. In addition, a drop of isopropanol (IPA) was applied to the sensor window prior to repositioning it on the skin to prevent dew from forming on the frozen tissue surface and interfering with the optical measurements.

The fiber array was set up horizontally, and light from each fiber was picked up by a photodiode in the detector unit. The depth of freezing was determined by analyzing the fluctuation of backscattered intensities measured during thawing and correlating it with transmission data obtained from the calibration procedure. To replicate body temperature, tissue samples were mounted on a heated platform. During data collection, the sensor head was in contact with the skin and allowed to thaw. In experiments using a hot plate, thawing took between 85 and 100 s, and the skin temperature rose from −40 °C to +20 °C. The procedure was repeated multiple times on various skin samples and portions, with similar results, varying mostly in the overall duration of thawing due to variations in the application of cryogen.

**Figure 2:**
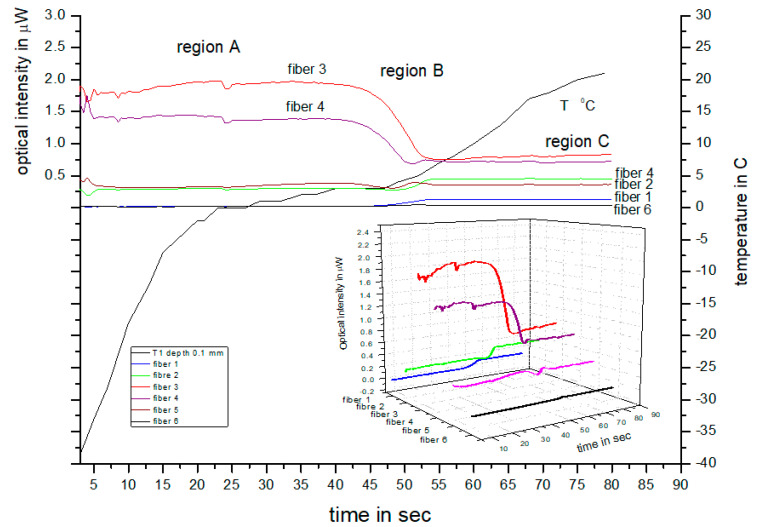
Timelines of intensities and temperature showing the temporal change in temperature and backscattering optical intensities from the six fibers during thawing [13].

Figure 2 depicts the typical backscattering intensity timelines measured by the six detection fibers, together with the temperature fluctuations measured by a thermocouple positioned less than 0.5 mm under the skin. This experiment was very similar to the one in [13] and was repeated in order to compare the porcine and in vivo measurements. As the sample thaws, the inner fibers 3 and 4 initially exhibit a constant intensity, which reduces as the temperature rises. However, the intensity timelines of the outer signal fibers 1, 2, 5, and 6 demonstrate an increase in signal strength that drops off as the sample thaws. The decrease in intensity toward the periphery is associated with an increase in backscattering, leading to a reduction in optical diffusion in the frozen tissue. Consequently, less light can reach the outer fibers, as illustrated more distinctly in the 3D graph shown in Figure 2. The thawing process is separated into three parts that are broadly dependent on the temperature variations, which initially change rapidly between −40 °C and −2 °C (region A). Similarly, the temperature gradient remains nearly constant around 0 °C due to the latent heat of thawing and remains constant until the ice melts (region A), increasing thereafter (region C). When observing the timelines of the inner fibers 3 and 4, it is apparent that the intensity of the light transitions from high to low in region B. This transition is directly related to the reduction in scatterers associated with the shrinking of the “ice ball”, as indicated by our calibrations. Therefore, based on the transmitted and backscattered calibration described above, the maximum optical detection depth in porcine tissue is approximately 1.5 mm [13]. Consequently, the fiber array can only detect changes in the backscattered intensity when the ice ball is reduced to less than 1.5 mm. As the ice ball decreases to a depth of less than 1.5 mm, the reduction in scatterers causes a corresponding transition from high to low intensities, as observed in region B. Finally, when all the ice melts, the signal in region C is weak, which is associated with normal tissue.

**Figure 3:**
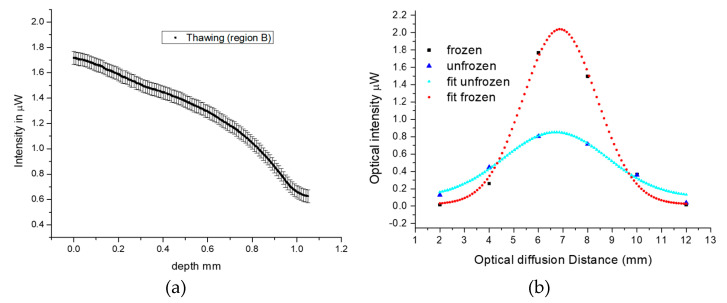
(**a**) Average backscattering intensity variation (of region B in Figure 2) with depth of freezing for the inner fibers 3 and 4 of the array sensor. (**b**) Optical diffusion in frozen and unfrozen tissues.

Based on this process, it is also possible to determine the change in backscattered intensity in relation to the depth of freezing, as shown in Figure 3a. This graph displays the average decrease in intensity of the inner fibers 3 and 4, recorded during the thawing process of multiple tissues under similar conditions. The associated errors are calculated based on the standard deviation of intensity and are approximately 5%.

Furthermore, the optical diffusion in frozen and unfrozen tissue can be determined by comparing the backscattering intensities detected by the six fibers during thawing, as determined by the data in the transition region. This can be seen in Figure 3b, where the known inter-fiber separation can be used to determine the optical diffusion by measuring the full width half maximum (FWHM) of the intensities across the six fibers for the frozen and unfrozen tissues. In the frozen tissue, the FWHM is approximately 3.5 mm, while in the unfrozen tissue, this extends to nearly 5 mm, indicating that light penetrates less in frozen tissue due to higher backscattering, which is consistent with the results shown in Figure 2.

## 4. Spectral Study and Comparison of Porcine and Human Spectra

It is possible to assess the depth of freezing during thawing of porcine ex vivo tissue based on our previous results. Due to the difficulty in calibrating the procedure with transmitted light, this method is not suitable for in vivo measurements. However, as described previously, during in vivo cryotherapy, there is a temporary change in the tissue’s color due to the formation of ice crystals and a reduction in blood flow, causing the skin to become temporarily pale and white. To quantify the spectral characteristics during this transition, in vivo skin measurements were performed during cryotherapy in the dermatology clinic. Our aim was to compare the spectral characteristics of thawing porcine ex vivo tissue with those of living human tissue in order to determine, semi-empirically, the frozen depth.

**Figure 4:**
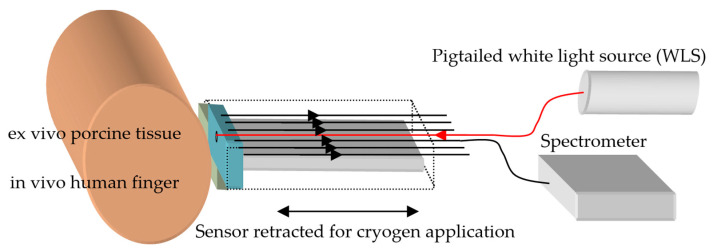
Schematic diagram of experimental setup used for spectral analysis of ex vivo porcine and in vivo human measurements.

The experimental setup is shown in Figure 4. To illuminate the tissues, a white light source (WLS) with the peak wavelength centered on 650 nm, consisting of a fiber-pigtailed light-emitting diode (LED) coupling approximately 0.5–0.8 mW of light to the tissue, was used. This peak wavelength was chosen to avoid interference with the intensity variations from oxy-hemoglobin for the in vivo experiments and also so that the results could be compared with the ex vivo results described earlier. The emission bandwidth of the WLS ranged from 350 nm to 900 nm. The backscattered spectrum from the tissues was coupled to the spectrometer by one of the inner fibers 3 or 4, located adjacent to the source fiber, and the data were stored in a computer.

**Figure 5:**
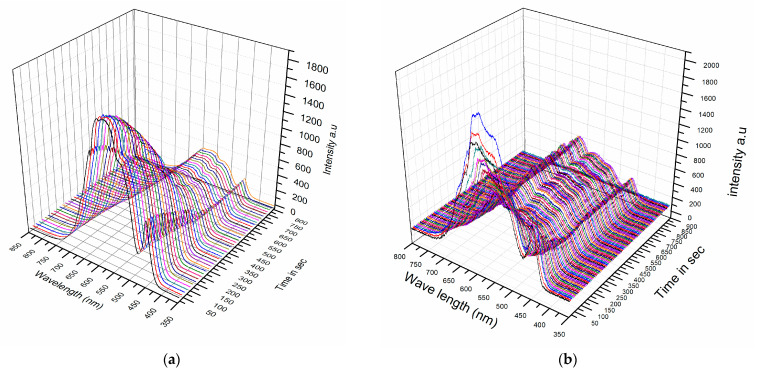
(**a**) Ex vivo porcine spectral measurements taken during thawing. (**b**) In vivo human finger spectral measurements taken during thawing.

The experimental procedure for measuring porcine ex vivo and in vivo human skin tissue (finger) involved preparing the sensor by lightly wiping the window with IPA to eliminate frozen dew, applying cryogen to the tissues, and placing the sensor in contact with them. Because in vivo human tissue thaws rapidly, measurements had to be obtained immediately after freezing. To simplify data acquisition, the sensor was mounted on a precision sliding stage. The ex vivo porcine tissue was held vertically on a stationary frame, as described in Section 3.1, without using a hot plate. In vivo cryosurgery was performed on warts growing on fingers by applying cryogen and subsequently positioning the sensor on the finger. Spectral measurements were taken with an Avantes (Avaspec 3648), collecting spectral data every 5–10 s during thawing. To avoid degradation of the ex vivo tissue, several freeze–thaw cycles were performed at different locations on the porcine tissue surface, and the spectral variations with time are shown in Figure 5a. Similarly, for the in vivo experiments on human fingers, the results were accumulated over several treatments using the aforementioned protocol, and typical results are shown in Figure 5b. It should be noted, however, that the spectral spike appearing on all spectra at around 425 nm is an artifact of the LED and is of no biological significance. Furthermore, the WLS was chosen due to low emissions above 800 nm, which is the absorption band of oxy-hemoglobin and could contribute to unwanted spectral variations related to heartbeats [14].

A comparison of the spectral responses between the two cases shows that they are similar, with the backscattering peak appearing at approximately 550–650 nm and overall spectral intensities higher during freezing and lower during thawing. However, the rate at which the overall spectral intensity decreased during thawing was much slower in the ex vivo porcine tissue compared to the in vivo human tissue. This difference was expected since the porcine tissue lacked a blood supply and thawing was mainly influenced by the ambient air temperature, whereas in living tissue, blood flow contributes to temperature equilibration. Additionally, the changes in peak intensity at 650 nm exhibit a similar temporal response to the porcine cases in Figure 2, with a transition region and two fixed states observed in both cases—one when the tissue is frozen and the other when it is thawed.

**Figure 6:**
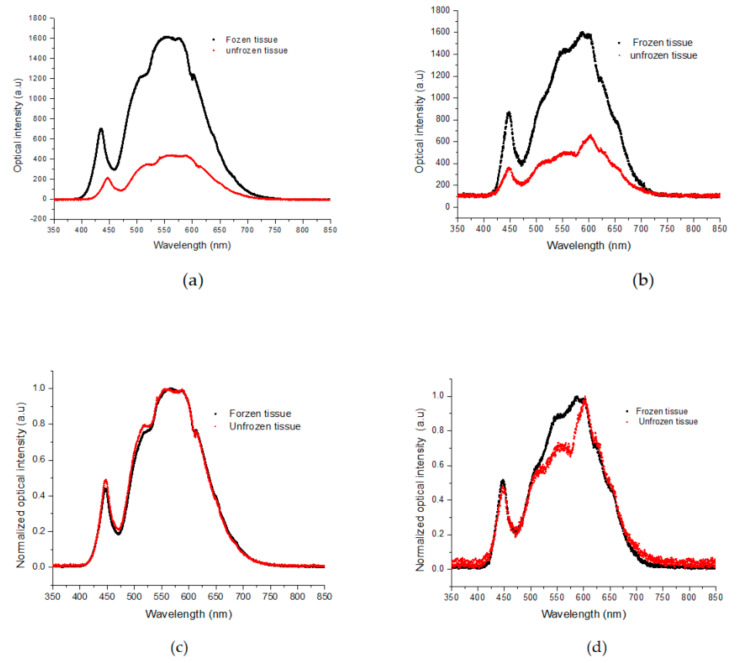
(**a**) Spectral variations in frozen (black) and unfrozen (red) porcine tissue. (**b**) Spectral variations in frozen (black) and unfrozen (red) live human finger tissue. (**c**) Normalized spectral variations in frozen (black) and unfrozen (red) porcine tissue, (**d**) Normalized spectral variations in frozen (black) and unfrozen (red) live human finger tissue.

The spectra of frozen and unfrozen tissues were plotted in Figure 6 to compare the ex vivo and in vivo spectral responses. The frozen spectra are shown in black, while the unfrozen ones are shown in red. At first glance, these spectra appear to be similar. However, when the spectra are normalized (min.–max. normalization) and plotted together, as shown in Figure 6c,d, with the frozen spectra in black and the unfrozen ones in red, the porcine ex vivo tissue shows the same variations. In contrast, the spectrum for the live human frozen/unfrozen tissue in Figure 6d exhibits spectral variations around 500 nm and 625 nm, which are attributed to the hemoglobin absorption peak [15].

**Figure 7:**
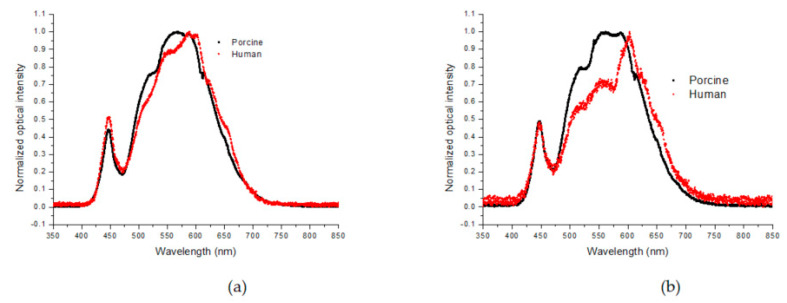
Comparison of frozen and unfrozen porcine/human tissues (black/red, respectively). (**a**) Frozen porcine/human tissue; (**b**) Unfrozen porcine/human tissue (black/red, respectively).

Based on the above results, it would be interesting to compare the frozen and unfrozen states of the two specimens to see if the calibration method from the study with porcine tissue can be used to measure the frozen depth in live human tissue. This is especially crucial as in vivo depth measurements of frozen-unfrozen tissues are not routinely feasible. To investigate the two specimens, the normalized spectra of their frozen-unfrozen states were compared, and the results are presented in Figure 7. Figure 7a shows the frozen porcine ex vivo and human in vivo spectra, which are very similar, indicating that backscattering from the ice in the tissues dominates. In Figure 7b, the normalized unfrozen spectra of the two specimens are shown, which are generally similar, with some spectral variations around 500–600 nm, associated with the absorption band of hemoglobin [14].

**Figure 8:**
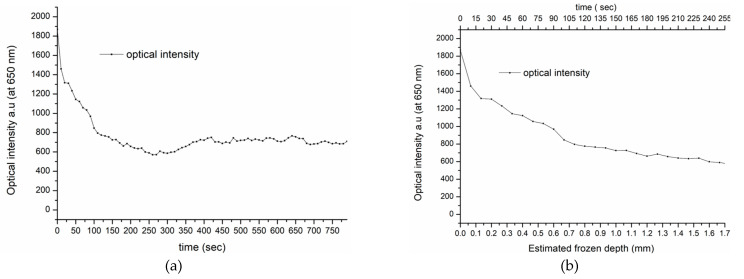
Thawing timeline of human tissues at 650 nm. (**a**) Total thawing timeline of human tissues. (**b**) Thawing timeline of initial 250 s of human tissue’s estimated frozen depth (mm).

Based on the spectral similarities of the frozen porcine and human tissues, we suggest that the depth of freezing in living human skin is similar to that in the porcine tissue and can be estimated at approximately 1.5 mm. If this assumption is correct, the thawing timeline for in vivo human tissue, taken at 650 nm from the spectra shown in Figure 5b, can be directly compared with the equivalent porcine specimen, shown in Figure 3a. Furthermore, at this wavelength, there is minimal interference from the intensity variations due to oxy-hemoglobin, associated with the heartbeat and the backscattering intensity at 650 nm, as shown in Figure 8a. Therefore, the transition from high to low intensity during thawing is assumed, as was seen in the porcine specimen, to correspond to the maximum frozen depth of 1.5 mm detected by the fiber sensor. This is shown in Figure 8b, which displays in more detail the intensity transition during thawing.

Consequently, Figure 8b indicates a similar process to that which determined the frozen depth measurement in the porcine tissue, as shown in Figure 3a. This finding provides an initial insight into the in vivo thawing process and its potential use in real-life cryotherapy applications.

## 5. Conclusions

We illuminated in vivo human skin tissues with white light and measured the backscattered spectrum with a fiber optic array sensor. The procedure involved applying cryogen to the tissue and measuring the spectral changes during thawing. The study found that the spectral responses from the porcine ex vivo tissue and the in vivo human tissue were similar, with a backscattering peak at approximately 550–650 nm and overall spectral intensities that were higher when the tissues were frozen and lower when they were thawed. However, the rate at which the overall spectral intensity decreased during thawing was much slower in the porcine tissue than in the in vivo tissue, conceivably due to homeostatic temperature regulation. We suggest that it is possible to semi-empirically determine the frozen depth of in vivo human tissue by comparing the spectral characteristics to those in thawing porcine ex vivo tissue.

## Data Availability

Data available on request.
